# Catastrophic health expenditures associated with single admission for paediatric surgery in a low-resource setting: a multicentre study

**DOI:** 10.1136/bmjgh-2025-022894

**Published:** 2026-07-16

**Authors:** Tunde Talib Sholadoye, Omolara M Williams, Olumide Abiodun Elebute, Okechukwu Hyginus Ekwunife, Felix Alakaloko, Lukman Abdur-Rahman, Deirdre Kruger, Elena Libhaber, Mwayi Kachapila, Christianah Oyegbola, Omotayo Adenike Oyewande, Aneel Bhangu, Ewen Munro Harrison, Dmitri Nepogodiev, James C Glasbey, Dion Morton, Emmanuel A Ameh, Adesoji O Ademuyiwa, CATERPILLAR study group, Adesoji Ademuyiwa, Adewale Adisa, Tunde Talib Sholadoye, Lukman Abdur-Rahman, Lofty-John Chukwuemeka Anyanwu, Usang E. Usang, Philemon Okoro, Abubakar Muhammad Auwal, Uchechukwu Ezomike, Taiwo Lawal, Okechukwu H. Ekwunife, Omolara Williams, Omolara Faboya, Olakayode Ogundoyin, Christopher Bode, Kefas John Bwala, Christianah Oyegbola, Omotayo Oyewande, Mwayi Kachapila, Raymond Oppong, Dhruva Ghosh, Dion Morton, Aneel Bhangu, Ewen Harrison, James Glasbey

**Affiliations:** 1 Surgery, Ahmadu Bello University, Zaria, Kaduna, Nigeria; 2 Ahmadu Bello University Teaching Hospital, Zaria, Kaduna, Nigeria; 3 Surgery, Lagos State University Teaching Hospital, Lagos, Nigeria; 4 Surgery, Lagos University Teaching Hospital, Surulere, Lagos, Nigeria; 5 Surgery, Nnamdi Azikiwe University Teaching Hospital Nnewi, Nnewi, Anambra, Nigeria; 6 Surgery, University of Ilorin Teaching Hospital, Ilorin, Kwara, Nigeria; 7 School of Clinical Medicine, Faculty of Health Sciences, University of the Witwatersrand, Johannesburg, South Africa; 8 Health Economics Unit, University of Birmingham, Birmingham, England, UK; 9 National Institute for Health Research, Global Surgery Unit, Lagos Hub, Lagos, Nigeria; 10 NIHR Global Health Research Unit on Global Surgery, Universities of Birmingham, Edinburgh and Warwick, Birmingham, UK; 11 Surgery, University of Edinburgh, Edinburgh, UK; 12 University of Birmingham, Birmingham, UK; 13 NIHR Global Health Research Unit on Global Surgery, University of Birmingham, Birmingham, UK; 14 Clinical and Experimental Medicine, University of Birmingham, Birmingham, UK; 15 National Hospital, Abuja, Abuja, Nigeria; 16 Division of Paediatric Surgery, National Hospital, Abuja, Nigeria, Abuja, Nigeria

**Keywords:** Surgery

## Abstract

**Background:**

Catastrophic healthcare expenditure (CHE) poses significant financial burdens on households globally, particularly in low- and middle-income countries. This study was designed to determine the prevalence of CHE among families of paediatric surgical patients admitted for surgery and to explore factors associated with it.

**Methods:**

The study was a prospective, multicentre, cross-sectional study carried out in 25 hospitals with paediatric surgery in Nigeria from July to November 2024. Consecutive patients below 16 years of age who underwent surgery were included. CHE was defined as health expenditure above 10% of the head of household annual household income. Data on demographic data, educational and employment status of parents and other related factors were collected and analysed using Stata.

**Results:**

A total of 1665 patients were recruited with ages ranging from 5 days to ≤16 years (median=4.74 years). Overall median annual income for the heads of household was US$1097.78. The median (IQR) cost of care was US$129.81 per patient and 56.3% of households spent out of pocket and only 11.7% of households had access to health insurance.

A total of 981 households (58.9%; 95% CI 56.5% to 61.3%) experienced CHE. The CHE incidence rate ratio (IRR) decreased among households where the patients were male (IRR 0.92 (95% CI 0.86 to 0.98); p=0.011), the head-of-households had a graduate/postgraduate education (IRR 0.64 (95% CI 0.53 to 0.78); p<0.001), but increased when major surgery was performed (IRR 1.25 (95% CI 1.08 to 1.44); p=0.002) and with prolonged hospital stay (1.008 (95% CI 1.005 to 1.011); p<0.001).

The main sources of funds for healthcare were out-of-pocket (n=936, 56.3%) and availability of health insurance was significant in preventing CHE, as three of every four households accessing health insurance did not experience it.

**Conclusion:**

Catastrophic health expenditure was highly prevalent among families of children who underwent surgery in Nigeria, particularly among those who had major and emergency surgeries.

**WHAT IS ALREADY KNOWN ON THIS TOPIC**

- More than half of Nigerians live in extreme poverty, and most household healthcare costs are out-of-pocket with a high risk of catastrophic healthcare expenditures.
- Nigeria’s very young population and the rising number of untreated paediatric surgical conditions, combined with increasing surgical and living costs, further exacerbate this financial vulnerability.
- Currently, most of the reports are limited to studies conducted in the southern part of Nigeria.

**WHAT THIS STUDY ADDS**

- This is the first national study to establish the prevalence of catastrophic healthcare expenditure (CHE) in families where a child needs surgery and the effect of the cost of surgery and admission on the household’s income in a country with low health insurance coverage and high out-of-pocket health expenditure.
- It showed the factors that influence catastrophic health expenditure and sources of households’ funds for health.

**HOW THIS STUDY MIGHT AFFECT RESEARCH, PRACTICE OR POLICY**

- The study showed the benefit of collaboration among surgical researchers to determine nationally representative data which can be used for planning interventions that will mitigate against such a high prevalence of CHE.
- Data from this study could also help shape policies that will prioritise paediatric surgical conditions in health insurance coverage, which at present is minimal in Nigeria and most low- and middle-income countries.

## Introduction

Catastrophic healthcare expenditure (CHE) is defined as out-of-pocket (OOP) expenditure exceeding 10% of a patient’s annual household income or 40% of annual household expenditure after food expenses.^1 2^ OOP expenditure is the payment made directly by a patient for healthcare services.^1 2^ The WHO estimates that 150 million people experience severe financial challenges annually, and about 100 million become poor or poorer as a result of healthcare expenditures.^3^

Worldwide, socio-economic inequality, household economic status, hospitalisation, presence of an elderly, disabled or person with a chronic illness in the family were the common significantly associated with household CHE.^4–9^ Furthermore, the prevalence of CHE also depended on the type of insurance scheme that the household had^4^; non-comprehensive insurance may result in a need to spend OOP and push the family into financial strain. CHE could lead to indebtedness, of which the burden rests heavily on the poor, with 90% occurring in low-income countries.^4 10 11^ Even where free paediatric surgical services were provided by the public sector, families continue to accumulate substantial OOP expenditure and a third of households incur catastrophic expenditure for a paediatric surgical procedure.^1^

In Nigeria, where 53.5% of Nigerians live on less than US$1.90 a day, there is an increased risk of CHE.^12–14^ Therefore, where incomes are low and prepayment mechanisms such as taxation and health insurance are lacking in healthcare financing, these households face a risk of incurring large healthcare expenditures when members fall ill.^5 6 9^ Nigeria is a ‘country of the young’ with almost half of the entire population under the age of 15 years, with an estimated 2.9 million children with surgically correctable conditions^15^ and at high risk of CHE in emergency gastrointestinal surgeries in tertiary hospitals.^16^ The health expenditure is highly subsidised through federal, state and local government health facilities amidst more accessible but usually more expensive private health facilities. Low health insurance coverage of <5% is the norm,^17^ usually national or state health insurance schemes but may not be comprehensive pushing most households to OOP health expenditure.^17 18^ The lack of health insurance coverage, particularly for children with surgical needs, puts them at a disadvantage. Smith *et al* recommended that investment in paediatric surgery in low- and middle-income countries by increasing financial risk protection mechanisms for families of children with surgical needs is essential to achieve national health goals.^19^

A recent survey observed an increasing number of untreated surgical conditions, such as hernia, undescended testes and trauma in children.^15^ With the rising incidence of paediatric surgical conditions among households in Nigeria brought about by population growth, coupled with the increasing cost of surgery, higher energy costs, economic changes^13 15 18^ and a setting where most households spend OOP for health expenditure,^16 18 20^ this predisposes these households to CHE. Therefore, this study was designed to determine the prevalence of catastrophic health expenditures associated with paediatric surgical procedures in Nigeria and factors associated with it. The findings from this study could serve as a veritable and objective reference to advocate for an expansion of national health insurance coverage to include children’s surgery.

## Methods

### Study design and setting

This was a prospective, multicentre, cross-sectional study of households whose children underwent surgical operations across hospitals with paediatric surgical expertise in Nigeria. Each participating hospital has a single research team made of surgeons involved in children’s surgeries, in addition to two or three collaborators including resident doctors, nurses and students. Each team collected data over a continuous 5-month period (from July to November 2024) at each participating centre to achieve the expected sample size. The selection of centres was by declaration of interest within the selection period (online supplemental table 8).

10.1136/bmjgh-2025-022894.supp2 Supplementary data

### Participants and definitions

All consecutive patients who presented to the centres and had elective and/or emergency children’s surgery were recruited into the study. There were no changes to routine patient management pathways in the institution. A household was defined as an entity/group comprising a child living with a parent(s)/guardian, and the head of the household is the parent/guardian who is responsible for the critical financial decisions in the family.

In this study, minor surgeries were surgeries that involved minimal tissue dissection and procedures lasting 1 hour or less, where the procedure was between an hour and 2 hours with significant tissue handling, but no resection of organs/tissue, or there were minimal haemodynamic changes, it is considered to be intermediate; other higher interventions were categorised as major surgery. Alternative funding is a source of funds besides OOP spending, including loans, charity and other sources of funds.

Catastrophic health expenditure was defined as health expenditure exceeding 10% of the head of household’s annual income (online supplemental appendix A).

The inclusion criteria were patients aged below 16 years who had any paediatric surgical procedure, either as planned (elective) surgery or emergency surgery. The exclusion criteria were patients with other comorbid conditions, such as sickle cell anaemia (due to need for special considerations in them), patients whose parents or legal guardians did not give consent, patients who had day case surgeries or patients whose surgeries did not require hospital admission, and patients who had multiple surgeries within a single admission. Patients whose parent(s) annual household income could not be calculated, and patients with incomplete data, were also excluded from the study.

The data were collected using validated case report form (CRF) (online supplemental appendix B)^21^ which included the patient biodata, diagnosis, surgical care timings and associated factors, surgery performed, outcome of surgery, source of funds for treatment (main source and others), direct healthcare cost (admission, investigation, anaesthesia, surgery, drugs and ward/intensive care unit (ICU) care, these were verified by review of receipts). Indirect medical costs (feeding, transportation and lodging; as informed by the parents), educational status of parents/heads of the household, employment status of the parents, monthly income of the parents (in Naira) and asset measurement for the International Wealth Index questions (online supplemental appendix B).

### Outcome measures

The primary outcome measure was CHE after paediatric surgical procedures.

The secondary outcome measure was the need for alternative funding mechanisms used for health expenditure.

### Data management and governance

Data were collected on paper CRFs and then entered into the Research Electronic Data Capture (REDCap) for data security.^21–23^ The service was managed centrally by the National Institute for Health Research, Global Surgery Unit Nigeria Hub. The data management and data security adhered to the requirements of the General Data Protection Regulations and the Nigerian Data Protection Act 2023.^24^ Collaborators were given secure REDCap project server login details, allowing secure data storage on the REDCap system (online supplemental appendix C).

All household incomes and costs of healthcare were obtained in Nigerian Naira (₦) but reported in both US dollars (US$) and Naira after conversion based on the prevailing average exchange rate from July to November 2024 (₦1617.81/US$). The National Principal Investigator coordinated the team and confirmed the accuracy of the data.

### Sample size determination

A systematic review in sub-Saharan Africa found that the incidence of CHE varies by threshold: on average, 23% of households experienced CHE when defined as health spending exceeding 10% of annual income, compared with 17% when the threshold was 40%.^25^ Based on this evidence, the study assumed a 20% CHE prevalence to calculate the sample size for a descriptive cross-sectional study. Using a 95% confidence level and a relative precision of 10% (absolute precision=0.02), the minimum required sample size was estimated to be 1537 patients.

Allowing for a 10% dropout or missing data rate, about 1700 households were required. The target sample size was achieved through 25 participating hospitals.

The study was conducted from a healthcare perspective, where only healthcare costs were measured (direct and indirect medical costs). While the categorisation of surgery into major or minor has been defined differently over the years,^26^ the simplicity made surgeons retain it in the surgical setting.

### Statistical analysis

A total of 1705 patients were recruited, and data on these patients and their households were collected. Forty households with incomplete data with no total cost of medical expenditure entered into the REDCap database were excluded from the final analysis (n=1665).

Statistical analyses of the collected data were performed using Stata V.17 suite of analytics software (StataCorp LLC, College Station, Texas, USA). The Shapiro-Wilk W test was used to determine the normality of continuous data. Descriptive statistics are presented as medians with IQRs for continuous data, and as frequencies with percentages for categorical data.

The relationship between various factors and the outcome of CHE was determined using the Mann-Whitney U test and Pearson’s χ² or Fisher’s exact test, as appropriate, for continuous and categorical data, respectively.

To identify factors associated with CHE, a mixed-effects Poisson regression was first performed. Because the rate of CHE was high, we estimated risk ratios using a modified Poisson regression with robust standard errors, as recommended by Zou.^27^ Logistic regression was avoided because ORs can overstate the true relative risk when the outcome is prevalent. Clinically plausible individual factors were included when building these regression models to predict CHE in paediatric patients. The hospital centre was included as a random effect in the model. As delay to surgery has a direct impact on length of hospital stay (LoHS), only the latter was included in the final model. For analysis purposes, only records with complete data were included in the final model (n=1584; 95.1%). Statistical significance was considered at a threshold p value <0.05.

### Patient and public involvement

The National Health Research Ethics Committee of Nigeria was involved in the initial review of the protocol, and the findings of the study shall be shared with the Ministry of Health and Social Welfare in Nigeria for dissemination of our findings and to guide policy change to reduce the risk of catastrophic health expenditure associated with children’s surgery.

## Results

A total of 1665 patients were included in the study. Overall, 599 (36.0%) were females and 1066 (64.0%) were males with a male-to-female ratio of 1.8:1. The age of the patients ranged from 5 days to 16 years and most patients (59.8%) were aged 1–11 years, with a median age of 4.74 (IQR 1.1–9.7) years or 56.9 (IQR 12.8–116.5) months. All hospitals are public hospitals at Federal (Teaching) or state (General) level tertiary institution, and are mostly urban-based (online supplemental table 8). Only 1.1% of study patients were admitted into a private ward or ICU, with 98.9% being admitted into open or general wards. Table 1 shows the demographic and clinical characteristics of the study population according to the CHE outcome. The household size of five and below in 896 (53.8%) households, six to eight in 528 (31.7%) households and nine and above in 240 (14.4%) households. The type of ward was not associated with CHE (p=0.233). Most of the patients were admitted for elective procedures (69.3%), of whom 54.9% experienced CHE (table 1). Conversely, CHE was significantly higher for emergency procedures at 68.0% (p<0.001).

**Table 1:** Demographic and clinical characteristics of the study population

Variable*	All patients n=1665	Catastrophic expenditure (CHE) n=981	No catastrophic expenditure n=684	P value
Age, median (IQR) in months	56.9 (12.8–116.5)	59.5 (9.9–120.8)	54.2 (15.3–111.5)	0.655
Sex, n (%)				
*Male*	1066 (64.0)	607 (56.9)	459 (43.1)	0.029
*Female*	599 (36.0)	374 (62.4)	225 (37.6)	
Timing of surgery, n (%)	n=*1663*			
*Elective*	1153 (69.3)	633 (54.9)	520 (45.1)	<0.001
*Emergency*	510 (30.7)	347 (68.0)	163 (32.0)	
Delay to surgery, n (%)	n=*1655*			
*Yes*	236 (14.3)	187 (79.2)	49 (20.8)	<0.001
*No*	1419 (85.74)	787 (55.5)	632 (44.5)	
LoHS, median (IQR) in days	9 (4–16)	12 (7–20)	5 (3–10)	<0.0001
LoHS >9 days, n (%)	756 (46.3)	585 (77.4)	171 (22.6)	<0.001
Complexity of surgery, n (%)	n=*708*			
*Major*	385 (54.4)	268 (69.6)	117 (30.4)	
*Intermediate*	260 (36.7)	139 (53.5)	121 (46.5)	<0.001
*Minor*	63 (8.9)	22 (34.9)	41 (65.1)	
Complications, n (%)	n=*1619*			
*Yes*	1474 (91.0)†	908 (61.6)	566 (38.4)	<0.001
*No*	145 (8.9)	60 (41.4)	85 (58.6)	

*Row percentages (%) are shown for categorical variables unless otherwise stated.

†77.8% (1147) of these were Clavien Dindo grade I.

CHE, catastrophic health expenditure; LoHS, length of hospital stay.

The study covered a variety of diagnoses, with the majority of patients presenting with gastrointestinal conditions requiring abdominal surgery (818, 49.4%), which was followed by genitourinary conditions (291, 17.6%). Specific common diagnoses included generalised peritonitis for bowel perforation (8.9%), inguinal hernia (5.3%), anorectal malformation (3.8%), hypospadias (3.8%), diseases of the appendix (3.4%) and Hirschsprung’s disease (3.2%) (online supplemental appendix D, tables 1.2). Surgical procedures performed included hernia repair (150, 9.0%), appendicectomy (62, 3.7%), urethroplasty (83, 5.0%), diagnostic laparotomies (2.0%), among other procedures (table 2).

**Table 2:** Surgeries performed among the study population

Surgery performed	All patients n(%)
Appendicectomy	62 (3.7)
Oesophagogastric surgeries	13 (0.8)
Excisional biopsy	100 (6.0)
Hepatobiliary surgeries	11 (0.7)
Hernia repair	150 (9.0)
Gynaecological surgeries	8 (0.5)
Herniotomy	28 (1.7)
Nephrectomy/upper tract surgeries	39 (2.3)
Orchidopexy	23 (1.4)
Colorectal surgeries/pull-through	89 (5.3)
Resection and anastomosis of bowel	118 (7.1)
Resection and enteric stoma formation	83 (5.0)
Reversal of enteric stoma	42 (2.5)
Urethroplasty	83 (5.0)
Other abdominal surgeries	109 (6.5)
Other lower urinary tract surgeries	56 (3.4)
Others	652 (39.1)
Total	1665 (100.0)

The median (IQR) cost of care was US$129.81 (US$80.5–210.39) (₦210 007.91 (₦129 500.00–340 371.00)) per patient. The median cost (IQR) of surgery was US$52.54 (US$34.00–88.01) (₦85 000.00 (₦55 000.00–142 386.00)). The total cost of care in emergency cases was significantly higher than in elective cases, with a median cost (IQR) of US$140.87 (₦227 900.89) (US$88.64–208.63) vs US$125.03 (US$74.92–211.55), respectively (p=0.045).

The median annual income of the father was US$1038.44 (US$445.05–1557.66) and the mother was US$370.87 (US$111.26–741.74). The median annual income of the family is US$1097.78 (US$519.22–1706.01), and 57.6% of the households earned less than US$1080 /year (table 3). A total of 981 (58.9%, 95% CI 56.5% to 61.3%) households experienced CHE for a single surgery. Age was not associated with CHE. Female sex of the patient was significantly associated with CHE (p=0.029).

**Table 3:** Socio-economic characteristics of the study population

Variable*	All patients n=1665	Catastrophic expenditure (CHE) n=981	No catastrophic expenditure n=684	P value
Level of education of HoH, n (%)				
*None/primary education*	285 (17.1)	212 (74.4)	73 (25.6)	
*Secondary/technical education*	520 (31.3)	361 (69.4)	159 (30.6)	<0.001
*Graduate/postgraduate*	859 (51.6)	408 (47.5)	451 (52.5)	
Employment status of HoH, n (%)				
*Full-time*	631 (37.9)	312 (49.5)	319 (50.6)	<0.001
*Other:*	1034 (62.1)	669 (64.7)	365 (35.3)	
*Part-time or self-employed*	*986* (*59.2*)	*627* (*63.6*)	*359* (*36.4*)	
*Unemployed*	*48* (*2.9*)	*42* (*87.5*)	*6* (*12.5*)	
Annual income for HoH in US dollar ($)†				
*Overall,* median (IQR**)**	1097.78 (519.22–1706.01)	667.57 (370.87–1112.62)	1483.49 (1112.62–2225.23)	
Originally in Nigerian Naira	1 776 000 (840 000–2 760 000)	1 080 000 (600 000–1 800 000)	2 400 000 (1 800 000–3 600 000)	
*According to employment status:*				
*Full-time*	1112.61 (741.74–1854.36)	741.74 (519.22–1186.78)	1483.49 (1112.62–2113.97)	
*Part-time or self-employed*	890.09 (445.05–1520.58)	630.48 (370.87–1 112.62)	1706.01 (1112.62–2966.98)	
*Unemployed*	322.66 (166.89–611.94)	322.66 (185.44–593.40)	222.52 (37.09–815.92)	

*Row percentages (%) are shown for categorical variables unless otherwise stated.

†Nigerian currency was converted to US$ based on the average exchange rate from July to November 2024.

CHE, catastrophic health expenditure; HoH, Head of Household.

Overall, 14.3% of study patients experienced a delay to surgery (table 1). Also, delays to surgery were experienced in 21.4% vs 11.1% of emergency versus elective patients, respectively, and 79.2% of patients who experienced a delay to surgery had CHE (p<0.001). The main reasons for delays were financial constraints (112, 47.5%), unavailability of surgical facilities (99, 41.9%), surgeon’s absence (24, 10.2%) and patient’s discharge against medical advice (3, 1.3%). The unavailability of surgical facilities was due to a lack of theatre space for emergency procedures (75, 75.8%), power outage (20, 20.2%), lack of anaesthesia gases (5, 5.1%) and lack of theatre scrubs (2, 2.1%).

Most patients required single procedures (n=1462, 87.8%) during the indexed surgery. The number of procedures performed per surgery also impacted CHE (pull-through vs pull-through plus protective colostomy). Each additional procedure increased resources used and healthcare costs substantially, and households in which patients underwent multiple procedures during the surgery were two to three times more likely to experience CHE compared with those with fewer procedures (p<0.001).

The complexity of surgery also significantly impacted CHE (table 1). Catastrophic healthcare expenditure was observed in 69.6% of major surgeries, 53.5% of intermediate surgeries and 34.9% of minor surgeries (p<0.001). Pairwise comparisons with Bonferroni correction remained significant for major versus intermediate surgery (adjusted p=0.003), major versus minor (adjusted p=0.003) and intermediate versus minor (adjusted p=0.024).

The occurrence of complications during treatment also significantly increased the likelihood of CHE (p<0.001), with 61.6% of patients with complications versus 41.4% of patients without complications resulting in CHE.

Table 3 shows the socio-economic characteristics of the study population. Households with a graduate/postgraduate level of education had lower CHE (47.5%) compared with those with primary/no education (74.4%) or secondary education (69.4%) (p<0.001). When the head of the household had full-time employment, this was associated with lower CHE (49.5%) versus part-time/self-employed/unemployed (64.7%) (table 3; p<0.001).

From table 4, the main source of funds for healthcare was OOP, and 53.4% of these households experienced CHE. Only 11.7% of patients relied on health insurance, and the smallest proportion of these households experienced CHE (27.2%). Online supplemental tables 3-6 showed complex relationship between source of funds and health insurance type.

**Table 4:** Funding sources for healthcare*

Source	All patients N	Percentage (95% CI)
Out of pocket	936 (56.3)	56.3 (53.9 to 58.7)
Charity	248 (14.9)	14.9 (13.3 to 16.7)
Loans	227 (13.7)	13.7 (12.1 to 15.4)
Health insurance	195 (11.7)	11.7 (10.3 to 13.3)
Sales of properties	57 (3.4)	3.4 (2.7 to 4.4)
Total	1663 (99.9%)	

*Most households use multiple sources of funds. This indicates the main sources of funding; missing, n=2. See online supplemental appendix D for the cross-table on the source of funds and health insurance (online supplemental tables 3-6).

292 patients (n=1627) had delayed discharge after surgery, 89 of which were due to financial constraints (16 of which were unable to pay the bill) and 70 due to complications of surgery (33 due to Surgical Site Infection). These increased the financial burden on the household and increased the chances of CHE (p<0.001)

As seen in table 1, the overall median (IQR) LoHS for the study population was 9 (4–16) days and longer LoHS was associated with a higher likelihood of CHE, with patients incurring CHE having a median (IQR) stay of 12 (7–20) days compared with 5 (3–10) days among those without CHE (p<0.0001).

Table 5 shows the univariate mixed-effects Poisson regression and multivariable mixed-effects Poisson regression with hospital centre included as a random effect for the prediction of CHE in this study population. In the univariate logistic regression analysis, being male, having a higher education level and full-time employment status were factors that reduced the odds of CHE, whereas a child scheduled for an emergency surgery, with a prolonged hospital stay and who underwent major surgery significantly increased the incidence rate of having CHE. The cost of consumables (40.3%) was a major contributor to direct cost resulting in CHE while the amount loss of parental working hours/days (47%) was major contribution to indirect cost (online supplemental table 7).

**Table 5:** Univariate and multivariable mixed-effects Poisson regression for the incidence of CHE (n=1584*)

Characteristics	Univariate mixed-effects Poisson regression		Multivariable mixed-effects Poisson regression	
	IRR (95% CI)	P value	Adjusted IRR (95% CI)	P value
Age, *years*	1.00 (1.00 to 1.01)	0.262	1.00 (0.99 to 1.00)	0.416
Male	0.91 (0.84 to 0.99)	0.025	0.92 (0.86 to 0.98)	0.011
Level of education of HoH (*vs baseline of primary/no education*)				
*Secondary/technical education*	0.82 (0.71 to 0.94)	0.006	0.86 (0.74 to 1.00)	0.051
*Graduate/postgraduate education*	0.59 (0.48 to 0.72)	<0.001	0.64 (0.53 to 0.78)	<0.001
Employment status of HoH (*full-time*)	0.81 (0.69 to 0.96)	0.013	1.01 (0.91 to 1.11)	0.906
Type of surgery (*emergency*)	1.21 (1.07 to 1.38)	0.003	1.12 (1.01 to 1.23)	0.028
Complications (*yes*)	1.36 (0.91 to 2.04)	0.134	1.36 (0.95 to 1.94)	0.096
LoHS, *days*	1.01 (1.005 to 1.015)	<0.001	1.01 (1.00 to 1.01)	<0.001
Complexity of surgery (*major†*)	1.40 (1.18 to 1.68)	<0.001	1.25 (1.08 to 1.44)	0.002

*Sample size was limited to patients having all observations for the included variables in the final model.

†Major (vs other=intermediate/minor).

CHE, catastrophic health expenditure; HoH, head of household; IRR, incidence rate ratio; LoHS, length of hospital stay.

However, on performing a multivariable mixed-effects Poisson regression analysis, the incidence rate of CHE decreased by 8% in males and by 36% in those households with a graduate/postgraduate education level, while increasing by 12% for emergency surgery, by ∼1% for every additional day of hospital stay and by 25% when undergoing major versus intermediate/minor surgery (p<0.0001).

However, on performing a multiple logistic regression analysis, the likelihood of CHE decreased by 23% in males and by 57% in those with a higher education level, while increasing by more than three times if the LoHS exceeded 9 days. Complication alone is not an independent predictor of CHE (table 5). Having complications and a delay in surgery increased the odds of experiencing CHE by 62% and more than doubled, respectively, after adjusting for age, employment status of the head of household and type of surgery (elective/emergency). This multivariate model (p<0.0001) showed goodness-of-fit and low collinearity.

## Discussion

A single household hospitalisation can trigger catastrophic health expenditure (CHE), as seen in this study where 58.9% of households experienced CHE.^28 29^ Although other studies have examined CHE in non-communicable diseases, this is the first national survey to assess CHE among children needing surgical care in Nigeria and sub-Saharan Africa. Existing paediatric surgery studies in the region focus on outcomes for abdominal sepsis,^16^ intussusception^18^ and day cases,^30^ mostly in southern Nigeria. Households with a sick or hospitalised child tend to have higher CHE,^2 5 6 9^ but such children were excluded from this study to isolate the specific effects of surgical care for non-complicated conditions on household spending

Total household income was comparable to the head of household’s income, as the head (father or mother) primarily bears spending responsibility—though dual-earning parents share the burden. Financial resources are not always fully pooled, and health expenditures are typically covered by the primary income controller.^31^ As such, the head of the household’s income more accurately reflects the resources available for healthcare spending decisions. Using total household income may overestimate available resources and underestimate the financial burden. Mothers earned considerably less than fathers, as has been reported by others^30^ and regardless of gender or employment status, heads earned under US$741.74 annually, risking CHE. Median incomes for fully employed or self-employed heads were similar, but other employment types varied widely; 40 households had no fixed income, relying on charity/loans. Lower income (<US$462) increases CHE risk,^32^ and income/socio-economic status are strong CHE determinants.^5 8 9^

While direct cost of health expenditure is the main factor for CHE, an unacceptably high incidence of CHE was observed even when the direct cost was controlled. The high incidence of CHE due to indirect costs of health expenditure was related to the loss of income following parental loss of work hours and the costs of feeding during admission. This was seen irrespective of accessibility to health insurance coverage.

Wealthy households were least likely to have CHE, though using private facilities raised CHE risk up to 10-fold,^29^ and travelling to big cities increased risk sevenfold.^33^ Despite most children having single surgeries, CHE was 58.9%. CHE risk is higher among the poorest quartile, rural, female-headed, uneducated, unemployed or uninsured households; this is similar to some reports.^2 5 6 9^ Conversely, a Togo study found higher CHE with male heads.^10^ In a report on intussusception, greater than 90% of uninsured households had CHE.^18^ This and our primary data confirm CHE remains high even with multiple funding sources.

Graduate-level education of the head significantly protected against CHE, while below-secondary education led to greater than 70% CHE. Higher education improves health-seeking and insurance access,^2 4 29 32^ though some studies found no significant association.^29 34^ At least secondary education offered some protection, likely due to better employment prospects, thus lower CHE risk.^4–6^

Full parental employment was seen to reduce CHE; two-thirds of heads without full employment had CHE, and this rose to 87.5% for unemployed and 100% for volunteers. It can then be stated that unstable income limits financial resilience. Full employment and well-established self-employment provided stable income and better insurance coverage, consistent with other studies.^4 32^

Few patients were admitted to private wards, which did not significantly affect CHE, though private facility use is associated with higher CHE risk (up to 10-fold),^2 5 29 33^ especially for poor households.^5 6^

Even among those with health insurance, funding sources are typically multiple due to coverage limits. OOP spending is the main source, as only a small proportion had insurance—a low rate consistent with other studies.^16 18 20^ More households were shown to rely on charity and loans than on insurance. Many combine insurance, OOP, charity, loans and even asset sales to cover costs. This unplanned expenditure significantly worsens household financial status.^8 9 29^ Prepayment schemes have been suggested to limit OOP’s impact.^6^ Low socio-economic households face financial hardship from healthcare expenses, pushing them below the poverty line and increasing CHE risk.^4 34^

Surgical delays stemmed from disease severity, added investigations or—most often—lack of funds. Financial constraint was the most common cause of delayed treatment. Delays create a ripple effect, increasing healthcare costs and facility use,^28 33 35 36^ leading to CHE that further impoverishes poor families already seeking alternative funds. Prolonged hospital stays (>11 days) carried a significant CHE risk. Presurgery or transfer delays raised costs, facility use and worsened outcomes.^35^ Key factors were financial burden and weak organisational capacity; operational changes to reduce delays would lower expenditure and improve outcomes.^35^

Discharge delays—due to unpaid bills, complications or institutional issues—also predispose households to CHE. Delayed discharge or repeat visits directly increase total care costs, raising CHE risk.^2 5 20 33^ Therefore, ineffective healthcare service delivery and hospital efficiency issues are contributory to the risk of CHE.

OOP spending and low health shock resilience^8 9 29^ can push households into CHE, undermining health goals and universal health coverage. In similar settings, ∼97% of households below the poverty line face OOP expenses that push them further below it.^3 6^

Even with a single surgery, households faced high CHE, as reported by several authors.^19 20^ Notably, some congenital anomalies require two to three surgeries in the same sitting, raising care costs. Households with such multiple surgeries were two to three times more likely to have CHE, as additional surgeries add costs at all levels and increase complication risk, particularly with complex anomalies.

Over half of patients had major surgery, with higher costs inherent to surgical complexity—fees are directly proportional to complexity and categorised as such by hospitals, mainly due to greater consumable use. Consumable cost was a major contributor to direct cost of health expenditure resulting in CHE in this study. Complex surgical diseases also require more investigations, visits and longer stays,^33^ characterising many complex congenital anomalies needing surgery. Surgical complexity is a strong CHE predictor, even after adjusting for emergency status, education and employment, since greater complexity demands more extensive preparations and resources. Though elective cases predominated, emergency surgeries significantly increased CHE risk, as emergency care bypasses financial planning, raising OOP costs.^16^ Delays associated with emergency surgery may result from late referrals or the patronage of alternative health facilities before seeking specialist care, associated with increased risk of complications that drive up CHE. Unplanned expenditure on surgical diagnosis and treatment predisposes households to CHE. Notably, while surgery is expensive, a recent study on non-communicable diseases found that medications and diagnostics were the main OOP drivers increasing care costs.^33^

The incidence of CHE was lower for male patients and for households with a higher-educated head. Differences in hospital characteristics and funding structures may influence costs and partly explain heterogeneity in CHE among centres.

Complications often prolong hospital stays, add surgeries and raise costs. In this study, although 61% of patients with complications experienced CHE, complications were not an independent predictor of CHE. This could be explained since very high complications were mainly of Clavien Dindo grade I and may not significantly increase the cost of care. One study found a fivefold cost increase for similar conditions when complications occur,^35^ and repeated postdischarge follow-up carried a 1.5-fold CHE risk.^2 5 33^

Prolonged hospital stay resulted from surgical delays, financial burden, complications and other factors. Length of stay increased household financial burden: households with CHE had a significantly prolonged stay of 12 days versus 5 days without CHE. With other factors controlled, each additional hospital day increased CHE incidence, consistent with a prior report.^20^ Longer stays raised costs for medications, diagnostics and staff services,^28 33^ and for resource-limited households, this may push them below the poverty line.^3^

### Limitations

The study focused on single surgery in patients who had in-hospital care excluding day care surgeries that are common with a lower risk of CHE and those with multiple surgeries in a single admission that may be due to complications and a higher risk of CHE.

The estimation of the income in categories other than fully employed heads of household is challenging and it may be difficult to eliminate reporting bias and the possibility of selection bias.

The cost of surgery also varies in major towns compared with small towns, and so it is with the level of the hospital, even when similar levels of care are given. This is a cross-sectional study whose findings are Nigeria-specific but may align with likely trends in other sub-Saharan nations.

### Policy recommendations

The policy implications of these findings are substantial. First, the low proportion of households with health insurance and the markedly lower CHE among insured families indicate that Nigeria’s current financing arrangements do not adequately protect children who require surgery. National and subnational policymakers should therefore expand financial risk protection for paediatric surgery through the National Health Insurance Authority and state insurance schemes, with explicit inclusion of common paediatric surgical procedures, emergency operations, diagnostics, anaesthesia, consumables, ward care and treatment of complications in benefit packages. Because many vulnerable households work in the informal sector, enrolment strategies should also extend beyond salaried workers through simplified family-based or state-supported pathways for low-income households.

Second, policymakers should consider subsidy mechanisms, exemption policies or special paediatric surgical funds for high-cost and emergency procedures, particularly for uninsured and socioeconomically vulnerable households. Prompt in-hospital assessment, fast-track review by medical social welfare teams and transparent package-based costing for common operations to reduce uncertainty and unplanned expenditure. Hospital managers should also improve theatre scheduling, emergency theatre access, procurement of consumables and discharge coordination, while governments should strengthen infrastructure and supply systems that reduce avoidable delays.^37^

Third, in this study, the indirect cost constitutes a major factor in the occurrence of CHE; these are mainly from parental loss of income due to loss of work hours and the feeding costs while on admission. Policies to provide the systems structure for wholly or partly subsidised feeding programmes for children on admission; and promoting paid parental leave in the formal and informal sectors may remarkably reduce the effects of indirect cost of care.

Fourth, there is data support the recognition of paediatric surgery as a child health, equity and poverty-reduction priority rather than a niche tertiary service. Integrating children’s surgical care into child health policy, universal health coverage reforms and hospital quality-improvement programmes would help ensure that access to surgery is not determined by a household’s ability to absorb sudden costs. For hospitals, practical steps include strengthening complication prevention, reducing delayed discharge due to unpaid bills and routinely monitoring time-to-surgery, length of stay and patient-level expenditure as service performance indicators. For policymakers, the findings provide empirical justification for financing reforms that move paediatric surgery away from dependence on OOP spending and towards pooled, equitable and predictable funding.^38^

## Conclusion

CHE is highly prevalent among households following paediatric surgery, particularly in emergencies, major operations, low-income and poorly educated families. Limited insurance coverage exacerbates financial vulnerability. The study showed the need to expand financial risk protection, strengthen health insurance and the reduction of treatment delays to safeguard children’s access to surgical care and advance universal health coverage. It also identified loss of parental work hours and in-hospital feeding as an important factor in occurrence of CHE.

10.1136/bmjgh-2025-022894.supp1 Supplementary data

## Data Availability

Data are available upon reasonable request.
