# Supplementary material for: Catastrophic health expenditures associated with single admission for paediatric surgery in a low-resource setting: a multicentre study
Source: BMJ Glob Health. 2026 Jul 16;11(7):e022894. doi: 10.1136/bmjgh-2025-022894 (PMC13384123; doi:10.1136/bmjgh-2025-022894)
Supplement: Supplementary data [file bmjgh-11-7-s002.pdf]

Supplementary

Appendix A: Definitions of Key Outcomes

A. Catastrophic Expenditure

Catastrophic health expenditure was defined as health expenditure exceeding 10% of a head of household's annual household income. The healthcare financing is usually out of pocket from the household budget, but it is mainly by breadwinner/head of the household. Female head of the household is functionally equivalent to female breadwinner.

B. Main source of funds is what contributes more than 60% of the total money, this is determined by asking the parent that is responsible for payment.

C. Alternative Sources of Healthcare Funding - National health insurance system - Private insurance, - Community-based insurance

- Thrift contributions

- Religious bodies

D.

Delayed Surgery refers to a planned surgical procedure that is not performed after diagnosis is made, instead postponed. The determination is surgeon specific.

Definition of CHE

CHE = OOP > 10/100 of Head of Household annual income (HoHai), where:

OOP = Indirect Medical Cost + Direct Medical Cost

HoHai = The annual income of parent (Father or mother) financing the medical bills

TAHI = Father's annual income + Mother's annual income

Appendix B: Summary of data fields (Case Report Form)[21]

**Patient Data Identification Fields**	**Required data (definition / comment)**
**1. Date of birth**	**Day/month/year**
**2. Patient sex**	**Male**<br>**Female**

Diagnosis and Surgery Details Data Fields

**3. Diagnosis**	**see table S2**
**4. Surgery performed**	
**5. Date of Surgery**	**(dd/mm/yyyy)**
**6. Timing of Surgery**	**Elective or Emergency**
**7. Delayed surgery**	**No**<br>**Yes (Reason if yes)**<br>- Surgeon's absence<br>- Unavailable surgical facilities<br>● Power outage<br>● Lack of anaesthesia gases<br>● Lack of theatre scrub<br>● Lack of theatre space (in the case of emergency)<br>- Patient's financial constraints<br>- Patient's discharge against medical advice
**8. Surgical complications according to Clavien-Dindo classification:**	**Grade I**<br>**Grade II**<br>**Grade IIIa**<br>**Grade IIIb**<br>**Grade IVa**<br>**Grade IVb**<br>**Grade V**
**9. Number of surgeries performed**	**1, 2, 3, 4, 5 and above**
**10. Length of stay in hospital**	
**11. Date of Discharge**	**dd/mm/yyyy**
**12. Delayed discharge**	**No**<br>**Yes**<br>**Reason for delayed discharge if 'Yes'**<br>- Slow recovery<br>- Unpaid hospital bills<br>- Others
**13. Type of ward admitted into**	**Open**<br>**Private**

Direct Healthcare Costs Data Fields

14. Total cost for admission (NGN)

15. Cost of surgery (including cost of anaesthesia, anaesthesia pack, surgery, and surgical pack, NGN)

16. Cost of surgical consumables (NGN)

17. Cost of laboratory investigations (NGN)

18. Cost of medications (NGN)

19. Cost of ICU (if used) (NGN)

20. Total cost of care (NGN)

In-direct Medical Costs Data Fields

**21. Father/Mother/Caregiver**	**Yes**<br>**No**
**22. Loss of working hours/days due to child's surgery**	

23. If yes, how many hours/days?

***24.* Amount lost due to less working hours/days (NGN)**

25. Cost of feeding during child's surgery

26. Cost of transportation during child's surgery

27. Cost of accommodation during child's surgery

Household Details Data Fields
Head of Household

**28. Gender of head of household**	**Male**<br>**Female**
**29. Level of education of head of household**	**Primary**<br>**Secondary**<br>**Graduate**<br>**Postgraduate**

	**Full-time**
**30. Employment status of head of household**	**Part-time**
	**Contract**
	**Casual**
	**Self-employed**
	**Volunteer**
	**Unemployed**
	**Professional**
**31. Occupational category of head of household**	**Managerial**
	**Skilled non manual**
	**Skilled manual**
	**Semiskilled manual**
	**Unskilled manual**
	**Lower**
**32. Socioeconomic status of head of household**	**Middle**
	**Lower**
**33. Average Monthly Income of Head of Household**	**0 – 50,000 ($0 - $30)**
	**51,000 – 100,000 ($31 - $60)**
	**101,000 – 150,000 ($61 - $90)**
	**151,000 – 200,000 ($91 - $120)**
	**201,000 – 250,000 ($121 - $150)**
	**251,000 – 300,000 ($151 - $180)**
	**301,000 and above ($181 and above)**
**Father/Mother/Caregiver**	
**34. Level of education**	**Primary**
	**Secondary**
	**Graduate**
	**Postgraduate**
**35. Employment status**	**Full-time**
	**Part-time**
	**Contract**
	**Casual**
	**Self-employed**
	**Volunteer**
	**Unemployed**
**36. Occupational category**	**Professional**
	**Managerial**
	**Skilled non manual**
	**Skilled manual**
	**Semiskilled manual**
	**Unskilled manual**

39. Average Monthly Income	**0 – 50,000 ($0 - $30)**<br>**51,000 – 100,000 ($31 - $60)**<br>**101,000 – 150,000 ($61 - $90)**<br>**151,000 – 200,000 ($91 - $120)**<br>**201,000 – 250,000 ($121 - $150)**<br>**251,000 – 300,000 ($151 - $180)**<br>**301,000 and above ($181 and above)**

40. Monthly household expenditure on food

41. Monthly income after deduction of food cost

***42.* Is the patient under any health insurance coverage**

**43. Household size**	**Small household (5 and below)**<br>**Medium household (5 to 8)**<br>**Skilled non manual (8 and above)**
**44. At least one member in the household has used a private healthcare facility**	**Yes**<br>**No**

Asset measurement for the International Wealth Index

**45. Availability of various consumer durables in household**	**Television**<br>**Refrigerator**<br>**Car**<br>**Bike**<br>**Bicycle**<br>**Utensils**<br>**Housing Characteristics**<br>**Input number**
**46. Housing Characteristics**	

Funding and financial distress strategies

**47. Source of funds for current healthcare expenditure**	**Loan**<br>**Out-of-pocket**<br>**Charity**<br>**Sales of properties**<br>**Health Insurance**
**48. Source of health insurance if any:**	**Private Insurance**<br>**National Health Insurance Authority**<br>**State health insurance scheme**<br>**Community-based insurance**

**49. Sales of household items to fund healthcare expenditure**	**Yes**<br>**No**
**50. Source of the loan if gotten**	**Individual**<br>**Thrift contribution**<br>**Loan organization**<br>**Religious body**<br>**Extended family**
**51. Amount of loan (NGN)**	
**Others**	
**52. Type of Healthcare facility visited**	**Private**<br>**General**<br>**Teaching**
	**Rural**
**53. Healthcare Location**	**Semi-urban**<br>**Urban**

*It is surgeon specific.

Appendix C: Methodology

1. Criteria for centre inclusion within the CATERPILLAR study:

● Obtain all appropriate local approvals for the conduct of the CATERPILLAR study audit.
● Successful completion of data collection within the stipulated period at the centre. Individual data collection will only be included when:
i. >95% data completeness has been achieved.
ii. All data for the period has been uploaded within the specified deadlines.

2. Steps for successful inclusion of centres

1. The Steering Committee was informed about participation in the CATERPILLAR study.

2. The local team was formed, up to three collaborators. A Paediatric Surgeon should be co-ordinating the team and leading audit registration and data collection. The Surgeon should be supported by at least one motivated Resident doctor, and Nurse or medical student.

3. Confirmation of formal audit approval from the hospital's clinical audit department prior to commencing data collection. Ensure that the audit department knows that the study is part of a national project and that patients' data shall be enter data on REDCap.

Centres joined at different points due to ethical approval delays. Where necessary, the National ethical approval was shared with the local ethical committees to support local ethical application.

4. Obtain permission from the hospital's Guardian (or the audit department) to submit and store data to REDCap.

5. Submit the local ethical approval and permission to the country lead, Lagos Hub. REDCap accounts was then issued to the collaborators.

6. Local meetings addressed:

● Role assignment decision at each stage of the project (e.g. identifying patients, collecting baseline data, completing follow-up, data entry to REDCap).

● Rehearsal on patient identification and collect required data.

7. Identify all patients fitting the inclusion criteria within your specified 5-month window. Contact your regional lead with any questions or issues that may arise over your data collection period.

8. Ensure all data has been uploaded to the REDCap system by the data collection deadline, and completed all fields, avoiding missing data points. If more than 5% of patients at a centre have missing data, the centre cannot be included in the CATERPILLAR dataset.

9. It is a condition of participation in the CATERPILLAR that following completion of the audit at the centres, the local results were presented to the hospital's surgical department and/or reported back to the audit department.

3. Quality assurance:

Design: The protocol for the study was written with guidance from experts in a cross-speciality advisory group.

Training: The CATERPILLAR study Leads had local meetings with collaborating teams to brief them on the protocol, and feedback from any local issues or questions raised was addressed. Several training sessions for research assistants on the protocol and the CFR were done, and further issues were addressed as the study progressed.

Project team structure: At each centre, the study was delivered and disseminated by teams of Paediatric and other Surgeons, resident doctors, and medical students.

Data completeness: Following data collection, only data sets with >95% data completeness were accepted for pooled national analysis. All data that lacked clear monthly/annual household income data were excluded from the analysis.

Validation: The methodology of using Redcap has been widely validated across multiple datasets, demonstrating high levels of case ascertainment (typically greater than 90 to 95%) and data accuracy (between 96 to 98%).[20-22]Each data entry by the collaborators was verified and confirmed by the sites' Principal Investigators.

4. Authorship:

The research outputs from the CATERPILLAR study will be listed under The CATERPILLAR study group.

All collaborators will be listed as PubMed-citable collaborators by the roles defined below:

1. Steering Committee: A core group of Paediatric Surgeons who have overall responsibility for protocol design, project coordination, and data handling.
2. Writing Group: A group of Paediatric Surgeons and the Steering Committee members responsible for the overall scientific content, data analysis, and preparation of research manuscripts.
3. Statistical Analysis: A small team of dedicated statisticians takes overall responsibility for the statistical analysis plan and quality assurance of data analysis.
4. Centre Leads: A single lead point of contact for data collection at each site who has overall responsibility for site governance, registration and coordinating handover between local collaborator teams. Local Leads should be prospectively identified by NIHR GSU Nigeria Hub Management, and these are recommended to be Paediatric Surgeons within the hospital team, and only one person can fulfil this role. They are responsible for the centre coordination of the study, acting as a link between hospital teams and the steering committee. Requirements for authorship on the CATERPILLAR study outputs include:
    a. Active engagement with the dissemination of the CATERPILLAR study and other NIHR GSU Nigeria Hub activities in their centres.
    b. Effective and responsive communication with the NIHR GSU Nigeria Hub steering committee, and with local collaborators throughout their time as centre Leads.
    c. Responsible for representing NIHR GSU Nigeria Hub at regional educational and research meetings.
    d. The primary person responsible for obtaining local approvals for the conduct of the CATERPILLAR study audit.
    e. Active involvement in a team during a data collection period at the centre, which meets the criteria for inclusion within the CATERPILLAR study dataset.
    f. To coordinate handover between all local collaborator teams at the centre and be involved in local dissemination of the CATERPILLAR study activities.
    g. Presentation of local results from the CATERPILLAR study audit at their centre (or otherwise arrange for another collaborator to present on their behalf).
5. Local collaborators (data collectors): A team of up to 3 people for small hospitals, and 5-12 people for larger hospitals, will be responsible for data collection per speciality group

over the specific 5-month period at a particular centre. This should ideally be formed by a Paediatric Surgeon, a Resident doctor, and a medical student/Nurse. The number of collaborators per team will be adjustable according to the number of patients included, up to a maximum of 12 collaborators in case there is inclusion of more than 100 patients. (1 collaborator for every 15 patients recruited) Minimum requirements for authorship on the CATERPILLAR study outputs include:

a. Compliance with local audit approval processes and data governance policies.
b. Active involvement in data collection over at least one data collection period at a centre which meets the criteria for inclusion within the CATERPILLAR study dataset (below).
c. While assistance with other teams is encouraged, collaborator status will only be assessed based on the successful completion of the allocated period.
d. Collaboration with the local lead to ensure that the audit results are reported back to the NIHR GSU Nigeria Hub.

5. **Dissemination of findings:** It was shared with all members of the caterpillar study group. The findings of the study were presented at the 23rd and 24th APSON conference in Ilorin and Abuja, Nigeria, the NIHR conference in Lagos and the PAPSA conference in Abidjan. It shall be published in a high-impact journal. Parents of patients who were interested in the findings will get a summary of the findings. The Ministry of Health and Social will get a summary of the findings and recommendations to influence policy for children's health.

Appendix D: Other related data

Table S1. The regions related to the diagnosis of the study population

Diagnostic Category	All patients n (%)
Abdomen	818 (49.4)
Chest	20 (1.2)
ENT	76 (4.6)
Genitourinary	291 (17.6)
Head & Neck	69 (4.2)
Neurological	105 (6.3)
Musculoskeletal	90 (5.4)
Other	83 (5.1)
Skin & Soft Tissue	38 (2.3)
Tumour	65 (3.9)
Total	1656 (99.5%)

Table S2. The detailed diagnosis of the study population

Diagnosis	Frequency(Percent)
ABDOMEN - Abdominal wall defects (Gastroschisis)	8(0.5)
ABDOMEN - Abdominal wall defects (Omphalocele)	3(0.2)
ABDOMEN - Abdominal wall defects (PBS, Cloacal anomalies, etc)	7(0.4)
ABDOMEN - Adnexal tumors	3(0.2)
ABDOMEN - Anorectal malformation	63(3.8)
ABDOMEN - Appendicular diseases	57(3.4)
ABDOMEN - Biliary atresia	8(0.5)
ABDOMEN - Diaphragmatic hernia	1(0.1)
ABDOMEN - Duodenal atresia	3(0.2)
ABDOMEN - Gallstones	1(0.1)
ABDOMEN - Gastroesophageal reflux disease (GERD)	1(0.1)
ABDOMEN - Hiatal hernia	4(0.2)
ABDOMEN - Hirschsprung's disease	53(3.2)
ABDOMEN - Hypertrophic pyloric stenosis	6(0.4)
ABDOMEN - Inflammatory bowel disease (IBD)	1(0.1)
ABDOMEN - Inguinal hernia	88(5.3)
ABDOMEN - Intestinal atresia (Jejunal, ileal)	2(0.1)
ABDOMEN - Intestinal obstruction	37(2.2)
ABDOMEN - Intussusception	57(3.4)
ABDOMEN - Malrotation and volvulus	15(0.9)
ABDOMEN - Pancreatic problems (Cysts/pseudocysts)	2(0.1)
ABDOMEN - Peritonitis	148(8.9)
ABDOMEN - Pyloric stenosis	1(0.1)
ABDOMEN - Rectal prolapse	3(0.2)
ABDOMEN - Ulcerative colitis	1(0.1)
ABDOMEN - Umbilical hernia	33(2.0)
ABDOMEN - Wilms tumor	5(0.3)
CHEST - Congenital heart disease (surgical aspect)	2(0.1)
CHEST - Corrosive oesophageal injury/stricture	5(0.3)
CHEST - Empyema	3(0.2)
CHEST - Esophageal atresia and tracheoesophageal fistula	2(0.1)
CHEST - Mediastinal masses	1(0.1)
CONGENITAL - Arteriovenous malformation	1(0.1)
GENITOURINARY - Bladder exstrophy -epispadias complex	6(0.4)
GENITOURINARY - Bladder outlet obstruction	7(0.4)
GENITOURINARY - Disorder of sexual development (46XX,46XY, Ovotesicular, gonadal disgenesis, etc)	7(0.4)
GENITOURINARY - Hydrocele	30(1.8)

GENITOURINARY - Hypospadias	64(3.8)
GENITOURINARY - Posterior urethral valves	16(1.0)
GENITOURINARY - Preputial pathology/diagnosis	4(0.2)
GENITOURINARY - Undescended testis (cryptorchidism)	24(1.4)
GENITOURINARY - ureteric anomalies	2(0.1)
GENITOURINARY - Ureteropelvic junction (UPJ) obstruction	2(0.1)
GENITOURINARY - Urinary stones	7(0.4)
GENITOURINARY - Vesicoureteral reflux (VUR)	1(0.1)
GENITOURINARY - Wilms' tumor	3(0.2)
GENITOURINARY- Cystic disease of the kidney	2(0.1)
GENITOURINARY-testicular torsion	3(0.2)
HEAD & NECK - Branchial cleft cyst	3(0.2)
HEAD & NECK - Cleft lip and palate	28(1.7)
HEAD & NECK - Congenital neck masses	4(0.2)
HEAD & NECK - Lymphatic malformations	3(0.2)
HEAD & NECK - Pediatric thyroid disease	1(0.1)
HEAD & NECK - Thyroglossal duct cyst	10(0.64)
NEONATAL - Neonatal bowel obstruction	1(0.1)
NEUROLOGICAL - Chiari malformations	2(0.1)
NEUROLOGICAL - Hydrocephalus	39(2.3)
NEUROLOGICAL - Neural tube defects (Spina bifida)	8(0.5)
ORTHOPEDIC - Clubfoot (talipes equinovarus)	2(0.1)
ORTHOPEDIC - Limb length discrepancy	1(0.1)
ORTHOPEDIC - Osteomyelitis	7(0.4)
ORTHOPEDIC - Pediatric fractures	11(0.7)
ORTHOPEDIC - Slipped capital femoral epiphysis (SCFE)	1(0.1)
Other	698(41.9)
OTHER - Cancer	1(0.1)
OTHER - Foreign body ingestion/aspiration	2(0.1)
OTHER - Germ cell tumors	1(0.1)
OTHER - Hyperhidrosis	1(0.1)
OTHER - Pediatric solid tumors (neuroblastoma)	1(0.1)
OTHER - Pediatric solid tumors (specified)	1(0.1)
OTHER - Pediatric trauma	8(0.5)
OTHER - Sacrococcygeal teratoma	4(0.2)
OTHER - Vascular anomalies	2(0.1)
SKIN & SOFT TISSUE - Burns	7(0.4)
SKIN & SOFT TISSUE - Hemangiomas	2(0.1)
SKIN & SOFT TISSUE - Soft tissue sarcomas	4(0.2)
Total	1665(99.5)

Table S3. The sources of health insurance among households with health insurance

Source of health insurance	Frequency(%)
	1170 (70.3)
Community-based insurance	96(5.8)
National health insurance authority	208(12.5)
Private Insurance	143(8.6)
State health insurance scheme	48(2.9)
Total	1665(100)

Table S4. The sources of loan among households with had loan

Source of loan	Frequency(%)
	1425(85.6)
Extended family	34(2.0)
Individual	138(8.3)
Loan organization	45(2.7)
Religious bodies	5(0.3)
Thrift contribution	18(1.1)
Total	1665(100.0)

Table S5. The Source of fund for current healthcare by source of health insurance among households

		Source of health insurance					Total
			Community-based insurance	National health insurance authority	Private Insurance	State health insurance scheme	
Source of fund for current healthcare		2	0	0	0	0	2
	Charity	201	18	3	22	4	248
	Health Insurance	3	1	159	17	15	195
	Loan	163	24	12	15	13	227

	Out-of-pocket (income)	770	34	32	88	12	936
	Sales of properties	31	19	2	1	4	57
Total		1170	96	208	143	48	1665

Table S6: Showing the complex nature of source of funds for current healthcare against the source of loan if gotten

		Source of loan						Total
			Extended family	Individual	Loan organization	Religious bodies	Thrift contribution	
Source of fund for current healthcare		2	0	0	0	0	0	2
	Charity	247	0	1	0	0	0	248
	Health Insurance	193	0	2	0	0	0	195
	Loan	1	34	124	45	5	18	227
	Out-of-pocket (income)	927	0	9	0	0	0	936
	Sales of properties	55	0	2	0	0	0	57
Total		1425	34	138	45	5	18	1665

Table S7. Major drivers of direct and indirect costs

**Cost Component**	**Mean Cost (USD)**	**Median (IQR) (USD)**	**Proportion of Total Cost (%)**	
	**Direct Costs (CHE incidence = 58.9%)**			**Proportion of Direct Cost (%)**
Total cost for admission	23.22	9.27 (3.09 – 21.63)	8.0%	13.5%
Cost of surgery	31.81	18.54 (9.27 – 39.56)	10.9%	18.5%
Cost of surgical consumables	69.06	52.54 (34.00 – 88.01)	23.7%	40.3%
Cost of laboratory investigations	22.15	12.36 (3.71 – 27.82)	7.6%	12.9%

Cost of medications	23.32	12.36 (6.18 – 24.72)	8.0%	13.6%
Cost of ICU (if used)	2.77	0 (0–0)	1.0%	1.6%
**Total Direct Costs**	171.54	129.81 (80.05 – 210.16)	59.1%	
	**Indirect Costs (CHE incidence=37.0%)**			**Proportion of Indirect Cost (%)**
Amount lost due to less working hours/days	55.96	12.36 (0–55.63)	19.2%	47.0%
Cost of feeding during child's surgery	43.69	25.34 (9.89–55.38)	15.0%	36.7%
Cost of transportation during child's surgery	15.53	9.27 (3.71–18.54)	5.3%	13.0%
Cost of accommodation during child's surgery	3.86	0 (0–0)	1.3%	3.2%
**Total Indirect Costs**	119.03	63.05 (26.58–137.84)	40.9%	

Table S8. Distribution of patients per type of healthcare facility and location

**Healthcare facility type**	**Rural**<br>**n (%)**	**Semi-urban**<br>**n (%)**	**Urban**<br>**n (%)**	**Total**<br>**n (%)**
General Hospital	-	-	75 (100.00)	75 (100.00)
Teaching Hospital	47 (2.96)	288 (18.11)	1,255 (78.93)	1,590 (100.00)
**Total**	47 (2.82)	288 (17.31)	1,330 (79.87)	1,665 (100.00)
**Senatorial distribution of hospitals**	Number of participating hospitals		States Involved	
**North central**	4		FCT Abuja, Kwara, Kogi	
**North east**	3		Bauchi, Borno, Gombe	
**North west**	5		Kaduna, Kano, Katsina, Kebbi,Jigawa	
**South east**	4		Abia, Anambra, Ebonyi, Enugu	
**South south**	3		Cross river, Edo, Rivers	
**South west**	6		Lagos, Ondo, Ogun, Osun, Oyo	
