# Supplementary material for: Catastrophic health expenditures associated with single admission for paediatric surgery in a low-resource setting: a multicentre study
Source: BMJ Glob Health. 2026 Jul 16;11(7):e022894. doi: 10.1136/bmjgh-2025-022894 (PMC13384123; doi:10.1136/bmjgh-2025-022894)
Supplement: Supplementary data [file bmjgh-11-7-s001.pdf]

# BMJ Global Health Author Reflexivity Statement

Adapted from Morton, B., Vercueil, A., Masekela, R., Heinz, E., Reimer, L., Saleh, S., Kalinga, C., Seekles, M., Biccard, B., Chakaya, J., Abimbola, S., Obasi, A. and Oriyo, N. (2022), Consensus statement on measures to promote equitable authorship in the publication of research from international partnerships. Anaesthesia, 77: 264-276. https://doi.org/10.1111/anae.15597

| | |
|---|---|
| **Study conceptualisation** | |
| 1. How does this study address local research and policy priorities? | Nigeria has an expanding young population with increasing surgical needs. In a country where more than half live in extreme poverty, the cost of surgical and in hospital care puts a huge financial burden on the household. Current evidence on cost of surgical care is limited to the southern part of the country and makes national policy decisions difficult.<br>This is the first national study to establish the effect of the cost of surgery and admission on the household income in Nigeria to determine the level of catastrophic health expenditure and sources of household funds for health. |
| 2. How were local researchers involved in study design? | The local researchers initiated the study and produced the initial study proposal which was reviewed and modified after several meetings with international colleagues to fine tune the study design |
| **Research management** | |
| 3. How has funding been used to support the local research team(s)? | The fund provided was used for the national and institutional ethical approvals, stipends for the research assistants for completed data, stationaries at national and institutional levels and dissemination of study initial findings at conferences. |
| **Data acquisition and analysis** | |
| 4. How are research staff who conducted data collection acknowledged? | Research assistants that included medical students and nurses were given stipends for all completed data uploaded on the NIHR Lagos Hub REDCap. Funds for internet access and communication were also provided. They were acknowledged in the manuscript. |
| 5. How have members of the research partnership been provided with access to study data? | All institutional leads and research members had access to the data on the Redcap until the date when the data was locked for editing. The data was extracted after certification of completion and was circulated through shared emails. |
| 6. How were data used to develop analytical skills within the partnership? | The team members for the statistical analysis met virtually to determine the analysis of interest and data was analysed as a team, shared via emails for verification and queries where resolved at subsequent meeting until the final analysis was obtained. The team consisted of trained statisticians and seasoned research analysts. |
| **Data interpretation** | |
| 7. How have research partners collaborated in interpreting study data? | The partners were involved in the interpretation of the results and contributed by giving their suggestions on how to appropriately present our findings. |

| | |
|---|---|
| **Drafting and revising for intellectual content** | |
| 8. How were research partners supported to develop writing skills? | The partners collaborated with the local team from the study conception to the approval of the final manuscript for publication. |
| 9. How will research products be shared to address local needs? | The research work preliminary findings were presented at conferences of the Association of Paediatric Surgeons of Nigeria and Pan-African Paediatric Surgical Association in 2025. Following publication of our findings, a policy statement will be submitted to the Ministry of Health and Social Welfare in Nigeria. |
| **Authorship** | |
| 10. How is the leadership, contribution and ownership of this work by LMIC researchers recognised within the authorship? | The authorship list was determined by the level of contributions. The last two names are senior members of the Nigerian team, who in addition to other contributions, helped in the coordination of the research |
| 11. How have early career researchers across the partnership been included within the authorship team? | Early career authors were encouraged to participate in the study and were included in the order of authorship based on their levels of contribution |
| 12. How has gender balance been addressed within the authorship? | The authorship is a fair mix of genders based on contribution. Others that were not qualified to be included in the authorship list were captured as collaborators. |
| **Training** | |
| 13. How has the project contributed to training of LMIC researchers? | The research assistants were trained on data collection and the use of the RedCap. The early career researchers were guided on research analysis using STATA and presentation of data. |
| **Infrastructure** | |
| 14. How has the project contributed to improvements in local infrastructure? | The fund provided was limited to administrative, stationary, internet and publication support. The project however set a ground for national collaboration and central collection of data via NIHR Lagos Hub RedCap account. |
| **Governance** | |
| 15. What safeguarding procedures were used to protect local study participants and researchers? | There were no changes in the routine management of participants. The data management and data security adhered to the requirements of the General Data Protection Regulations (GDPR) and the Nigerian Data Protection Act 2023. Collaborators were given secure REDCap project server login details, allowing secure data storage on the REDCap system. Each data entry by the collaborators was verified and confirmed by the sites' Principal Investigators. All patients had unique identification numbers (hospital numbers) that were used as a check to prevent duplication. Consent for participation and collection of data was taken. |
